# Updates in the Field of Submucosal Endoscopy

**DOI:** 10.3390/life13010104

**Published:** 2022-12-30

**Authors:** Tadateru Maehata, Yoshinori Sato, Yusuke Nakamoto, Masaki Kato, Akiyo Kawashima, Hirofumi Kiyokawa, Hiroshi Yasuda, Hiroyuki Yamamoto, Keisuke Tateishi

**Affiliations:** Division of Gastroenterology, Department of Internal Medicine, St. Marianna University School of Medicine, Kawasaki 216-8511, Japan

**Keywords:** submucosal endoscopy, third space endoscopy, peroral endoscopic myotomy (POEM), endoscopic submucosal dissection (ESD), submucosal tunnel endoscopic resection (STER), gastric peroral endoscopic myotomy (G-POEM), Zenker’s peroral endoscopic myotomy (Z-POEM), pocket-creation method (PCM), water pressure method

## Abstract

Submucosal endoscopy (third-space endoscopy) can be defined as an endoscopic procedure performed in the submucosal space. This procedure is novel and has been utilized for delivery to the submucosal space in a variety of gastrointestinal diseases, such as a tumor, achalasia, gastroparesis, and subepithelial tumors. The main submucosal endoscopy includes peroral endoscopic myotomy, gastric peroral endoscopic myotomy, Zenker peroral endoscopic myotomy, submucosal tunneling for endoscopic resection, and endoscopic submucosal tunnel dissection. Submucosal endoscopy has been used as a viable alternative to surgical techniques because it is minimally invasive in the treatment and diagnosis of gastrointestinal diseases and disorders. However, there is limited evidence to prove this. This article reviews the current applications and evidence regarding submucosal endoscopy while exploring the possible future clinical applications in this field. As our understanding of these procedures improves, the future of submucosal endoscopy could be promising in the fields of diagnostic and therapeutic endoscopy.

## 1. Introduction

Over the past decades, endoscopy has developed from being a diagnostic tool to a therapeutic tool. Gastrointestinal endoscopy has made great progress through the development of the endoscopic submucosal dissection (ESD) technique. Technical advances in endoscopy were inspired by the concept of natural orifice transluminal endoscopic surgery (NOTES) [1,2]. NOTES is a novel technique that involves the invasion of “the mucosa and submucosa to the muscle layer” with a flexible endoscope by making full use of the ESD technique. A major concern regarding NOTES was the safety of access to the peritoneal cavity and the secure closure of the entry point into these spaces. Sumiyama et al. developed submucosal endoscopy with a mucosal flap safety valve (SEMF), in which the peritoneal cavity could be accessed using a submucosal tunnel and the defect closed by using the mucosal flap [3]. This concern has been overcome with the introduction of the SEMF. Thus, the significance of the submucosal space as an operating field was realized. Submucosal endoscopy, also called third-space endoscopy, can be defined as an endoscopic procedure performed in the submucosal space or third space. This procedure is a novel operating field and has been applied in clinical procedures recently [4]. Submucosal endoscopy utilizing the SEMF technique has been utilized for delivery to the submucosal space and the peritoneal or mediastinal cavity in a variety of gastrointestinal diseases such as a tumor, achalasia, gastroparesis, and subepithelial tumors. This technique is divided roughly into ESD and peroral endoscopic myotomy (POEM). This article reviews the current applications and evidence regarding submucosal endoscopy while exploring the possible future clinical applications in this field.

## 2. Peroral Endoscopic Myotomy

Inoue et al. performed the first clinical POEM for the treatment of achalasia [5]. The endoscopic management of achalasia and non-achalasia spastic esophageal motility disorders has advanced with the introduction of POEM. This is because POEM has a theoretical superiority in that it does not injure the body surface, and the direction and length of the muscle layer incision can be set arbitrarily. Therefore, POEM has become the standard radical treatment for esophageal achalasia and related disorders as it is less invasive and has a higher curative effect than conventional therapeutic methods, such as laparoscopic Heller myotomy (LHM) [6,7]. For this reason, POEM is the most studied of the submucosal endoscopy procedures currently being performed [8,9,10,11]. The Chicago classification divides achalasia into three types based on the high-resolution manometry (HRM) findings. According to Japanese POEM guidelines, the treatment outcomes of POEM for the Chicago classification of type I and type II esophageal achalasia are similar to those of balloon dilation or laparoscopic surgery. On the other hand, POEM is better than balloon dilation or laparoscopic surgery for type III achalasia [12]. The POEM technique involves the following steps: the creation of a submucosal cushion, mucosal incision, submucosal tunneling, a selective circular or full-thickness myotomy, and the closure of the mucosal incision [12,13]. The distinctive characteristic of POEM is that the range of myotomy can be freely set because it is approached from the esophageal lumen. In fact, long myotomy over the full length of the abnormal contraction is required in cases such as diffuse esophageal spasm; however, a myotomy more than 25 cm from the cervical esophagus can be relatively easily performed in POEM. Thus, POEM is the treatment of choice for patients with dysmotility of the esophageal body, such as vigorous achalasia and diffuse esophageal spasm [14].

### 2.1. Efficacy of POEM

Several large case series and meta-analyses have demonstrated the high clinical efficacy of POEM in achalasia with short-term and medium-term results (Table 1). A large multicenter retrospective study of >1300 patients conducted in Japan reported that the efficacy of POEM was approximately 95% within 6 months to 1 year after POEM [15]. Another large study conducted in India demonstrated a 90.9% clinical success rate in 1 year [16]. Moreover, a large international multicenter study of 205 patients reported symptom relief rates of 98%, 98%, and 91% at 6 months, 1 year, and 2 years, respectively [17]. A prospective multicenter study conducted in Japan also reported a high efficacy rate of 97.4% [18]. Thus, POEM could be a curative treatment for most patients with achalasia, with durable results of up to at least 1 year after the procedure. Although there are limited studies about the long-term outcomes of POEM, especially comparing the long-term outcomes of POEM with those of conventional treatment methods, Teitelbaum et al. reported that POEM for the treatment of achalasia resulted in durable long-term successful palliation of symptoms among 83% of patients after 5 years without the need for reintervention [19].

According to the literature comparing POEM with conventional therapeutic methods, such as LHM and pneumatic dilation (PD), POEM was more effective than LHM and PD, and numerous studies have demonstrated excellent patient outcomes [20,21,22,23,24,25]. In a randomized clinical trial that compared POEM with PD as the initial treatment for patients with achalasia, POEM resulted in a significantly higher treatment success rate at 2 years [26]. Furthermore, the 5-year follow-up of this randomized controlled trial also shows that POEM has a higher long-term efficacy and a lower risk of major treatment-related complications than PD [27]. A comprehensive review suggested that POEM is equivalent to LHM for achalasia regarding cost efficiency, hospital length of stay, and the relief of dysphagia, with comparable side effects. According to a systematic review and meta-analysis that recently compared outcomes of POEM, LHM, and PD by evaluating 19 studies, including five randomized trials and 4407 patients, the dysphagia reduction rates and postoperative Eckhardt scores were significantly superior for POEM compared with LHM and PD [28]. POEM could potentially replace LHM as the standard therapy for achalasia. Moreover, POEM is applied as a treatment option not only among adults but also among pediatric patients with achalasia [29,30]. In a systematic review and meta-analysis about POEM for the treatment of pediatric achalasia, there was a significant reduction in the Eckardt score and lower esophageal sphincter pressure in POEM, and 93% of the patients experienced both short- and long-term improvement or the resolution of the achalasia symptoms after POEM [31]. On the other hand, the factors that accounted for technical difficulties in POEM were less experience with the technique, sigmoid-type esophagus, and short tunnels (<3 cm).

**Table 1:** Reports on the outcome of peroral endoscopic myotomy (POEM) in achalasia (large studies).

Study	Patients (N)	Median Follow-Up (year)	Previous Treatment (%)	Efficacy (%)	Adverse Events (%)	Reflux (%)
Shiwaku et al., 2020 [15]	1346	1	31	95.1 (3 m) 94.7 (1 y)	3.7	14.8
Shiwaku et al., 2019 [18]	233	1	21.9	97.1 (3 m) 97.4 (1 y)	10.3	54.2
Li et al., 2018 [32]	564	4.1	34.2	94.2 (1 y) 92.2 (2y) 91.1 (3 y) 88.6 (4 y) 87.1 (5 y)	6.4	37.3
Nabi et al., 2018 [33]	502	1.8	48.2	90.9 (1 y) 86.0 (2 y) 81.2 (3 y)	1.6	21.5
Zhang et al., 2018 [34]	318	2.3	40.1	95.7	2.5	35.8
Kumbhari et al., 2017 [11]	282	1	28.6	94.3	−	23.2
Nabi et al., 2017 [16]	423	1.7	46	94 (1 y) 91 (2 y) 90 (3 y)	4.5	28.3
Ramchandani et al., 2016 [35]	220	1	41.3	94 (6 m) 92 (1 y)	6.4	21.6
Inoue et al., 2015 [8]	500	3	39	91 (1 y) 88.5 (3 y)	3.2	16.8

### 2.2. Adverse Events of POEM

Adverse events associated with POEM include mucosal injury, pleural effusion, pneumomediastinum, emphysema, pneumothorax, pneumonia, and bleeding. Shiwaku et al. reported that adverse events occurred in approximately 4% of the 1346 patients who underwent POEM, half of whom had mucosal injuries, including five cases of perforation, but no patients suffered adverse effects requiring surgical treatment [18]. The most common delayed adverse event is gastroesophageal reflux disease (GERD) [6,11,18,19,26,27,31]. A concern regarding POEM is that it may result in high rates of iatrogenic GERD. LHM and POEM cause postoperative GERD due to an impairment of the natural antireflux mechanisms. Therefore, LHM requires fundoplication to prevent GERD. However, in the case of POEM, a fundoplication is not performed. Recent studies have focused on GERD after POEM in greater detail and have reported an alarming incidence rate of 40–60% for GERD after POEM [6,11,18,19,26,28,36]. In a multicenter collaborative retrospective study, within 6 months after POEM, 63% of patients had erosive esophagitis [15]. Two reasons are considered for the risk of GERD after POEM. First, the prolonged myotomy of the gastric wall. Second, the dissection of the collar sling muscle, which suspends the angle of His. In order to prevent GERD after POEM, the double-scope method was developed recently. The double-scope method can prevent the prolongation of the myotomy toward the gastric wall by observing the tip of the endoscope regarding the submucosal layer using the pediatric scope in the stomach [37,38,39]. Moreover, a consensus meeting was held to discuss the management and prevention of GERD after POEM and how to deal with GERD refractory to acid-suppressing medications based on published papers and the personal experiences of each expert [39]. According to the results of this consensus meeting, it was confirmed that most patients with GERD after POEM respond to proton pump inhibitor therapy, and fundoplication for refractory GERD is rarely needed.

## 3. Peroral Endoscopic Tumor Resection/Submucosal Tunnel Endoscopic Resection

Peroral endoscopic tumor resection (POET) and submucosal tunnel endoscopic resection (STER) are novel treatments involving endoscopic enucleation of subepithelial tumors of the upper gastrointestinal tract (Table 2) [40,41,42,43,44,45,46,47,48,49,50]. These were reported at the same time and are almost the same procedures, but POET is an improved therapeutic technique based on POEM, while STER is an improved therapeutic and ESD-inspired technique. The resection of subepithelial tumors using a variety of endoscopic techniques, such as endoscopic mucosal resection and endoscopic submucosal resection (ESD), has been described previously [51,52]. However, these endoscopic techniques are limited to subepithelial tumors arising from the muscularis mucosa or submucosal layer. Subepithelial tumors arising from the muscularis propria have basically been managed via surgical resection. Therefore, endoscopic resection, especially ESD for subepithelial tumors, has the risk of perforation, and it is difficult to close the defect. On the other hand, in POET and STER, the endoscopic resection of subepithelial tumors is possible without full-thickness perforation by the creation of a submucosal tunnel as a working space for tumor resection while maintaining mucosal integrity. Recently, endoscopic full-thickness resection (EFTR) has been reported as a feasible technique for subepithelial tumors arising from the muscularis propria [53,54]. Some studies have reported the efficacy and safety of POET or STER for the treatment of subepithelial tumors. In a recent systematic review and meta-analysis, high rates of en bloc resection were reported [55]. According to a report with a large number of patients (290 patients with 4 years of follow-up), POET or STER also showed no residual tumor, local tumor recurrence, or distant metastasis. A recent retrospective study compared the outcomes of STER, ESD, and thoracoscopic enucleation (TE) for esophageal subepithelial tumors. STER yielded the shortest duration of hospitalization and the lowest cost compared with other modalities [56]. STER showed superiority to TE regarding procedure times and tumor location. Moreover, the resections for tumors with STER were larger than those for tumors with ESD. STER was more effective for the resection of large subepithelial tumors than ESD. A study comparing POET to TE demonstrated that the procedure time and duration of hospital stay for POET were significantly shorter than those for TE [57]. In a retrospective study that compared EFTR to STER, although there was no significant difference in treatment outcome, EFTR yielded longer durations of hospital stay [58]. These results indicated that the use of a submucosal tunnel decreases the risk of gastrointestinal tract leakage and infection. On the other hand, these studies should be interpreted with caution in extrapolating them to all subepithelial tumors, as most of the pathological diagnosis was leiomyoma. If the subepithelial tumor has malignant potential, such as GIST, en bloc resection with an intact capsule is necessary to avoid recurrence because GISTs have a fragile capsule compared to other subepithelial tumors, and there is a risk of seeding if the capsule ruptures. It is imperative that GISTs be resected with the capsule intact, which requires advanced POET or STER techniques and should only be performed by specialized operators at experienced facilities. The indications for POET and STER are as follows: (i) the best location of the tumor (esophagus and gastric cardia), (ii) growth type (intraluminal and intramural growth type), and (iii) tumor size (<40 mm or with symptoms). According to recent studies, POET and STER could safely achieve a high rate of en bloc resection for upper gastrointestinal subepithelial tumors smaller than 40 mm. However, there are some limitations to POET or STER. The main factors that make en bloc resection impossible are the large size and irregular shape of the tumor [57,59]. In particular, as there is a size limitation for tumors that can be retrieved perorally and as it is performed in a limited working space, tumors larger than 40 mm are generally not indicated for POET or STER.

## 4. Gastric Peroral Endoscopic Myotomy/Peroral Endoscopic Pyloromyotomy

Gastroparesis is defined as delayed gastric emptying in the absence of mechanical obstruction. Its main symptoms include early satiety, postprandial fullness, nausea, vomiting, bloating, and abdominal pain [60]. In severe cases, it can lead to weight loss and malnutrition. A diagnosis of gastroparesis is made based on the presence of the aforementioned clinical symptoms, an examination based on normal upper endoscopy that rules out any mechanical obstruction, and a test proving impaired gastric emptying. The causes are idiopathic in 36%, diabetic-related in 29%, and postsurgical status (abdominal surgery) in 7.5% of patients [61]. Gastroparesis is divided into two main pathologies: (i) peristaltic disorder and (ii) pyloric spasm and relaxation disorder. A subset of patients may not respond to dietary interventions and medications. Although the first-line treatment of gastroparesis is symptomatic control, only a few medications and dietary modifications are available for symptomatic control, and approximately 30% of patients do not respond to conservative treatment [62]. In the United States, metoclopramide is the only medication approved by the Food and Drug Administration for the treatment of gastroparesis. However, its therapeutic effect is unknown, and adverse effects, such as tardive dyskinesia, are concerns. Erythromycin may also be used, but its effect is limited. These limitations of conservative treatments, such as medication, highlight the need for an alternate treatment option [60]. Alternatively, treatment may include local injection of botulinum toxin, endoscopic stenting, and laparoscopic pyloroplasty. However, the effect of local injection of botulinum toxin did not prove effective in randomized trials with local injection of physiological saline [63,64]. Currently, the American College of Gastroenterology guidelines does not recommend a local injection of botulinum toxin. Endoscopic stenting and laparoscopic pyloroplasty may be performed as alternative treatments. However, there are problems of deviation in the case of stent insertion and problems of invasion under general anesthesia in the case of pyloroplasty. With significant advancements in submucosal endoscopy in the last few years, peroral endoscopic pyloromyotomy (POP), also known as gastric peroral endoscopic myotomy (G-POEM), has been developed as a treatment option for gastroparesis using myotomy [65,66]. The treatment of gastroparesis via myotomy uses essentially the same treatment technique as POEM, and reports of its use are increasing. Khashab et al. reported the first human case of POP without any adverse event and demonstrated significant clinical improvements at 1 year of follow-up [66]. This novel procedure has gained worldwide acclaim due to its less invasive nature and promising outcomes. Most previous studies have demonstrated a 100% technical success rate for the procedure. Most studies have presented short-term follow-up data (3–6 months), and some studies have presented long-term follow-up data (12–18 months) [67]. In a systematic review, the clinical response rate was reported to be 83.9% after the procedures [68]. Most studies showed significant improvement in clinical symptoms, especially nausea and vomiting. G-POEM can be performed on a variety of patients with different types of gastroparesis. The rate of adverse events (postoperative bleeding, pyloric ulcer, and tension capnoperitoneum) has been reported to be 0–6.7% [69,70,71]. In a recently reported randomized and sham-controlled trial that included 41 patients with severe gastroparesis, symptomatic improvement at 6 months was achieved in 71% of the patients after G-POEM compared with 22% after the sham procedure. Even more noteworthy is the fact that 75% of the patients achieved symptomatic improvements 6 months after cross-over G-POEM, which was offered to patients without treatment success after the sham procedure [72]. G-POEM is technically feasible in patients with gastroparesis. G-POEM has yielded excellent data regarding technical success rate, short-term outcomes, and adverse events. However, most data on G-POEM or POP were derived from nonrandomized studies with short-term outcomes. Therefore, the predictors of clinical and long-term outcomes need to be investigated further.

## 5. Diverticular Peroral Endoscopic Myotomy/Zenker’s Peroral Endoscopic Myotomy

Esophageal diverticula are rare sac-like outpouchings, and the prevalence rate is approximately 3% [73,74]. Zenker’s diverticulum (ZD) is the most common type of esophageal diverticulum. Most patients with esophageal diverticula are asymptomatic; however, they may experience dysphagia, regurgitation, and chest pain with disease progression. Treatment for esophageal diverticula should be considered for symptomatic cases, regardless of the size of the diverticulum. Although surgical treatment with cricopharyngeal myotomy is basically applied to esophageal diverticula, surgical treatment has high invasiveness and risk of adverse events. Following advancements in endoscopic techniques, endoscopic treatment for esophageal diverticula has become a widely accepted alternative to surgery. Conventionally, endoscopic septotomy has been applied to the esophageal diverticula. A meta-analysis of studies demonstrated that endoscopic septotomy yielded a significantly shorter procedure time and duration of hospital stay and fewer adverse events compared with the surgical group [75]. On the other hand, the rates of symptom recurrence after endoscopic septotomy were much higher than those after surgery due to incomplete septotomy. Submucosal tunneling septotomy by diverticular peroral endoscopic myotomy (D-POEM) is a novel technique and has been recently established as an effective method for complete septal dissection using a submucosal tunneling approach. Moreover, this technique has been applied effectively among patients with Zenker’s diverticulum (Z-POEM) [76,77,78]. In a large international study, the overall technical success rate was 97.3%, adverse events (such as bleeding and perforation) occurred in 6.7%, and the rate of symptom recurrence was 3.2% [79]. Moreover, a recent systematic review comparing Z-POEM and PerOral endoscopic septotomy (POES) also showed no difference in terms of both efficacy and safety [80]. A recent multicenter retrospective study demonstrated the overall technical success rate of D-POEM to be 90.9% without adverse events, clinical success to be achieved at 100%, and good short-term outcomes [81]. D-POEM and Z-POEM appear to be safe and feasible treatments for patients with symptomatic esophageal diverticula. On the other hand, the recent case report showed recurrence after Z-POEM despite complete septotomy [82]. The authors concluded that the extension of the esophageal myotomy was incomplete beyond the base of the septum, leading to a recurrence of the symptoms. This indicates that Z-POEM is not yet fully established procedurally. There has been a report that endoscopic treatment is also applicable in patients with previous surgical failure or clinical relapse, so there is a need to examine the possibility of retreating the use of endoscopic treatment in cases of recurrence [83]. There could be alternatives to direct endoscopic septotomy for ZD and minimally invasive treatment for patients with esophageal diverticula. However, no studies regarding D-POEM or Z-POEM have evaluated patients with medium- or long-term follow-up. Multicenter studies of D-POEM and Z-POEM with larger numbers and long-term follow-up are needed to further validate the safety and feasibility of D-POEM and Z-POEM.

## 6. Submucosal Tunneling Biopsy

The recent development of SEMF [3] is applied not only for use in the treatment of tumors but also for tissue sampling for diagnostic purposes [84,85,86]. This technique can be applied to access the muscular layer and myenteric plexus of the gastrointestinal tract less invasively for pathological diagnosis in patients with gastrointestinal motility disorders and to obtain biopsies of the submucosal tumors of the gastrointestinal tract. Particularly, esophageal motility disorders have unknown etiologies with various possible underlying pathologies and are currently defined only by manometry patterns. There are some techniques for the tissue sampling of the muscular layer of the gastrointestinal tract, but they have some limitations [87,88]. Precise histological diagnosis requires adequate tissue samples. However, endoscopic full-thickness biopsy has a high delayed perforation rate and is limited by a lack of adequate sample size. For subepithelial tumors, endoscopic ultrasound-guided fine-needle aspiration (EUS-FNA) is a safe and effective method, and it is the current standard modality for sampling gastrointestinal subepithelial tumors [89,90]. Nevertheless, in a systematic review with a meta-analysis, the diagnostic accuracy rate (59.9%) was moderate, and failures of FNA occurred due to insufficient samples for histological analysis, technical issues, location, and lesions smaller than 2 cm [91]. When considering the limitations of the aforementioned techniques, the submucosal tunneling method is a safe and effective method to perform a biopsy with direct visual control over the region of interest.

## 7. Endoscopic Submucosal “Tunnel” Dissection/Pocket-Creation Method

**Figure 1:**
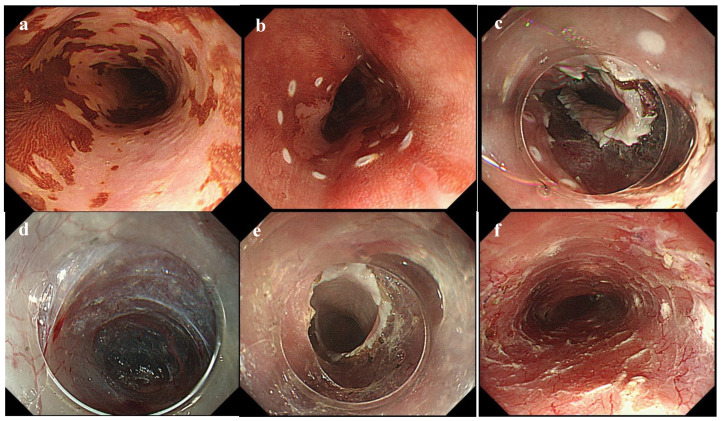
Endoscopic submucosal tunnel dissection for a circumferential superficial esophageal neoplastic lesion. (**a**) Chromoendoscopic image with iodine staining. (**b**) Circumferential markings were performed. (**c**) A complete circumferential mucosal incision was made to the distal side of the lesion to make an endpoint. (**d**) A submucosal tunnel was created. (**e**) The submucosal tunnel was created from an oral-to-anal incision through submucosal dissection. (**f**) The artificial ulcer after en bloc resection of the lesion.

**Figure 2:**
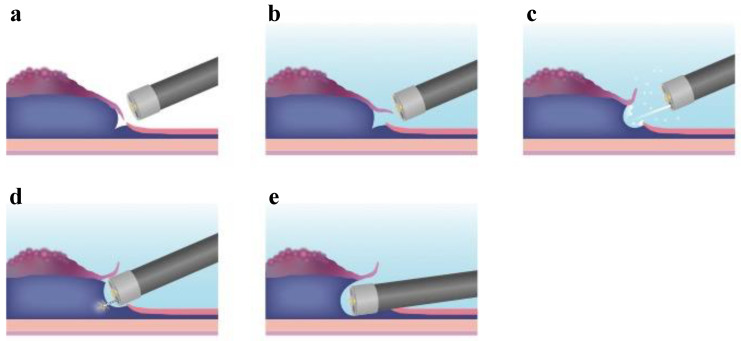
Methodologic schema of the water pressure method. (**a**) Partial mucosal incision is performed. (**b**) In an underwater situation, the opening of the mucosal flap is easier due to buoyancy. (**c**) Water pressure using the water-jet function of the endoscope helps with the insertion of the endoscope under the mucosal flap. (**d**) Approach of the endoscope to the submucosal layer becomes easy. (**e**) As a result, the water pressure method can be used to insert the endoscope quickly and easily into the submucosal layer.

Generally, one of the factors contributing to the technical difficulty of ESD is the lack of traction. Hence, several novel methods and strategies have been invented and developed to overcome technical difficulties. Endoscopic submucosal tunnel dissection (ESTD) [92,93] and the pocket-creation method (PCM) [94] are derived from ESD. These methods are new in the field of submucosal endoscopy, including POEM for achalasia and STER for submucosal tumors. These novel techniques are used to improve the ESD procedure without extra devices or equipment (Table 3). The creation of the tunnel in ESTD and PCM enables the tip of the endoscope to stabilize in the submucosal tunnel, providing sufficient traction even with severe submucosal fibrosis. Moreover, the advantage of creating a submucosal tunnel is the ability to maintain a clear view of the submucosal layer, in which efficient submucosal dissection becomes possible and contributes to shortening the procedure time. In ESTD, mucosal incisions are initially made at the anal and oral sides of the lesion after the submucosal injection, and then the submucosal dissection is performed between the anal and oral incisions to create a tunnel underneath the lesion. On the other hand, during PCM, a partial mucosal incision is first made on either the oral or anal side of the lesion in order to slip into the submucosal layer. Next, the middle aspect of the submucosal layer underneath the lesion is dissected to create a submucosal pocket. After creating the submucosal pocket, the mucosal incision is extended segmentally from the edges of the pocket. This method not only maintains a stable scope position inside the pocket with good traction but also prevents injection leakage. This approach also allows for tangential scope access even in situations in which the device often faces the muscle layer perpendicularly. ESTD is useful in the management of esophageal lesions due to a narrow lumen. Therefore, ESTD was developed as an alternative technique to esophageal ESD, particularly for circumferential superficial esophageal neoplastic lesions (Figure 1). The indications of ESTD for esophageal neoplastic lesions are lesions with a diameter of >20 mm and those involving at least one-third of the esophageal circumference. A recent meta-analysis revealed favorable short-term outcomes. The rates of en bloc resection, R0 resection, and curative resection were 98% (95% CI: 95.8–99.0%), 87.0% (95% CI: 78.2–92.5%), and 87.6% (95% CI: 67.4–96.0%), respectively [95]. In a propensity-matching analysis of conventional ESD for superficial esophageal squamous cell carcinoma, the ESTD group demonstrated a significantly shorter procedure time and submucosal dissection time than the conventional ESD group. In addition, ESTD reduced the rate of injury to the muscular layer [96]. On the other hand, PCM could be applied to other locations in the gastrointestinal tract, such as the colorectum. This is because the colorectal lumen is wide, and PCM can dissect not only the middle part of the submucosal layer underneath the lesion but also the lateral part, creating a pocket. PCM is advantageous in that it can decrease the leakage of the injection solution because the tunnel entry is made on either the oral or anal side. Therefore, PCM is useful for wide lesions such as laterally spreading tumors. A recent retrospective study revealed higher rates of en bloc resection and completed resection in the PCM group compared with the conventional ESD group. In addition, the dissection speed in the PCM group was faster than that in the conventional ESD group, while there was no significant difference in adverse events [97]. Among patients with severe fibrosis, PCM yielded a higher en bloc resection rate and a shorter mean procedure time than the conventional non-PCM method, and PCM reduced the discontinuation rate [98]. Recently, saline-pocket endoscopic submucosal dissection (SP-ESD) has been developed as a modification of PCM by filling the pocket with saline (saline immersion ESD), like underwater ESD. Water immersion facilitates the visualization of the dissection line because the endoscopic view in water immersion is clearer because of the refractive index of water. In a randomized controlled trial, the median dissection speed was significantly higher, and the median procedure time was significantly shorter in the SP-ESD group than in the conventional ESD group [99]. The insertion of a small-caliber-tip transparent (ST) hood under the mucosal flap is a crucial step in creating a submucosal tunnel. Therefore, we have developed a new technique, the “water pressure method” (Figure 2) [100]. Water pressure, induced by the water-jet function of the endoscope, facilitates the insertion of the ST hood under the mucosal flap. The “water pressure method” is simple and useful for ESD.

## 8. Solutions for Submucosal Injection

A solution for submucosal injection is essential to have good visualization and access to the submucosal space, not only for ESD but also for submucosal endoscopy. The ideal injection solution should be inexpensive, readily available, nontoxic, as well as safe, and efficient with a long-lasting submucosal cushion. Currently, various types of submucosal injection solutions have been developed in different countries. The representative solution is normal saline (NS). NS has been the most widely used because it is inexpensive, readily available, and nontoxic. However, the major limitation of NS is its rapid absorption into the surrounding tissues, which shortens the duration of an adequate submucosal cushion. Glycerol (Glyceol, Chugai Pharmaceutical Co., Tokyo, Japan), which is a hypertonic solution consisting of 10% glycerin and 5% fructose in an NS, is widely used as a submucosal injection solution in Japan. Because glycerol is hypertonic, it can take a longer submucosal elevation than NS. Moreover, glycerol causes no tissue damage, is inexpensive, and is readily available in Japan. Several studies have reported the usefulness of sodium hyaluronate (SH) as the longest-lasting submucosal elevation. Therefore, it is commonly used as one of the standard submucosal injection solutions in ESD. However, the main disadvantages of SH are its high cost and unavailability in Western countries. Eleview^®^ is a synthetic solution, including water for injection, medium-chain triglycerides, poloxamer 188, polyoxyl-15-hydroxystearate, sodium chloride, and methylene blue. Some studies have reported that Eleview^®^ is comparable to SH regarding submucosal elevation. Moreover, Eleview^®^ is a ready-to-use injectable liquid ampule. On the other hand, Eleview^®^ has high costs as an SH. We would normally use NS or Glyceol, and in difficult fibrotic situations, we would prefer to use SH. In any case, we should take into account the lesion features and the availability and costs of the solution, as well as the balance between its advantages and potential adverse effects.

## 9. Conclusions

Submucosal endoscopy derived from ESD is a novel operating field in which several new endoscopic procedures have emerged. Submucosal endoscopy has evolved over approximately a decade since the first report on POEM in 2010 and has been expanding into the field of the entire gastrointestinal tract. However, its efficacy, as assessed via long-term follow-up, remains unclear. Thus, further prospective large-scale studies with long-term follow-up are warranted to confirm the efficacy of submucosal endoscopy. In the near future, submucosal endoscopy holds the promise of a breakthrough in the diagnosis and treatment of gastrointestinal diseases and disorders.

## 10. Expert Opinion

Submucosal endoscopy has emerged as a novel operating field for interventional endoscopy, and its use has significantly increased over the past decade. The main reason is that submucosal endoscopy adopts the concept of SEMF. SEMF has enabled endoscopists to safely utilize the submucosal space. SEMF has become a breakthrough in the diagnosis and treatment of diseases that, to date, have an unknown region. POEM is an established initial treatment modality for achalasia using the submucosal space. The emergence of the concept of POEM in the submucosal space has dramatically evolved not only the treatment but also the diagnosis of diseases. Subsequently, POET/STER has been developed for the excision of subepithelial tumors from the esophagus and stomach. The other indications for submucosal endoscopy include refractory gastroparesis and ZD. G-POEM/POP and D-POEM/Z-POEM have been developed for patients with refractory gastroparesis. Moreover, ESTD/PCM was derived from ESD for the management of early gastrointestinal cancer. Recently, the submucosal tunneling technique has been applied not only in treatment but also in diagnosis. Submucosal tunneling biopsy has recently emerged. All submucosal endoscopic procedures use a similar technique to the submucosal tunneling technique. The submucosal tunneling technique is yet to be established and validated, and therefore, submucosal endoscopic procedures are likely to become popular in the near future. Although submucosal endoscopy is largely safe, and previous studies seem to yield promising results regarding all submucosal endoscopic procedures, some issues need to be resolved to facilitate these procedures. Regarding efficacy, evidence is lacking and limited for the majority of these procedures. It is possible that problems, such as adverse events, will surface with more time and the accumulation of data, such as from GERD after POEM. In particular, randomized studies and long-term follow-up data are yet to be accumulated, except those regarding POEM. There is a pressing need for additional meaningful data that can appropriately position these procedures. Therefore, as these procedures become increasingly common, it is necessary to address the issues of training, and further guidelines will be necessary. These procedures are not easy to learn as there are differences between Asia and other regions, even in ESD skill levels. Submucosal endoscopy is a complex procedure based on surgical principles. Nevertheless, these procedures are performed by not only surgeons but also endoscopists. Therefore, it is necessary to construct optimum training systems such as animal models and observerships at expert centers. In particular, endoscopists have to perform the procedure in close contact with surgeons when treating patients. After all, submucosal endoscopy is a less invasive procedure than surgery but is still somewhat invasive to the patient. Hence, the indications for these procedures must be decided strictly, and it is necessary to create guidelines. In the future, the concept of “inside and outside of the gastrointestinal tract” will lose practicality in endoscopic treatment, and it will become possible to diagnose and treat gastrointestinal diseases and disorders with an optimum approach and with minimal invasion while freely moving in the lumen and peritoneal cavity. Thus, it may be useful for investigating diseases for which the cause is unknown. In addition, improved devices and techniques may reduce procedure-related complexities and allow the endoscopist to perform these procedures more easily. As our understanding of these procedures improves, the future of submucosal endoscopy could hold promise in diagnostic as well as therapeutic endoscopy.

## Figures and Tables

**Table 2:** Reports on the outcome of peroral endoscopic tumor resection (POET)/submucosal tunnel endoscopic resection (STER) in subepithelial tumors (recent studies).

Study	Patients (N)	Location	Size (Range) (mm)	En Bloc Resection Rate (%)	Adverse Events (%)	Recurrence (%)	Pathological Diagnosis
Du et al., 2019 [48]	165	Esophagus: 106 Cardia: 59	20.0 (5.0–80.0)	77.6	21.2	0	Leiomyoma: 157 GIST: 3 Other: 5
Li et al., 2017 [47]	74	Esophagus: 74	18.9	98.6	9.5	2.7	Leiomyoma: 67 GIST: 7
Mao et al., 2017 [46]	56	Esophagus: 18 Stomach: 38	18 (10–32)	100	15.3	0	Leiomyoma: 45 GIST: 10 Other: 1
Chen et al., 2016 [44]	290	Esophagus: 199 Esophagogastric junction: 68 Stomach: 23	21 (10–70)	89.3	23.4	−	Leiomyoma: 226 GIST: 53 Other: 11
Wang et al., 2015 [43]	80 (tumors: 83)	Esophagus: 67 Cardia: 16	23.2 (10–55)	97.6	8.75	0	Leiomyoma: 68 GIST: 15
Ye et al., 2014 [42]	85	Esophagus: 60 Cardia: 16 Stomach: 9	19.2 (10–30)	100	9.4	0	Leiomyoma: 65 GIST: 19 Other: 1
Onimaru et al., 2020 [50]	47	Esophagus: 31 Cardia: 16	25.7	91.9	4.7	0	Leiomyoma: 34 GIST: 6 Other: 7
Chiu et al., 2019 [49]	51	Esophagus: 11 Stomach: 39 Duodenum: 1	20.71	94.1	4	1.96	Leiomyoma: 20 GIST: 15 Other: 16

**Table 3:** Clinical outcomes compared between PCM/ESTD and conventional ESD.

Study	Method	Patients Study/ESD (N)	Location	Specimen Size Study/ESD (mm)	Specimen Area Study/ESD (mm^2^)	Procedure Time (Study/ESD) (min)	Dissection Speed Study/ESD (mm^2^/min)	En Bloc Resection Rate Study/ESD (%)	Adverse Events Study/ESD (%)
Zhang et al., 2019 [101]	ESTD	32/55	Stomach	−	1573.0/930.1 (*p* < 0.01)	87.3/136.7 (*p* < 0.01)	18.0/7.8 (*p* < 0.01)	100/87.3 (*p* = 0.035)	59.4/100 (*p* < 0.01)
Zhang et al., 2018 [102]	ESTD	52/98	Esophagus	15.37/12.95	−	93.21/92.39	21.54/16.10 (*p* = 0.002)	96.15/88.78	9.62/8.16
Huang et al., 2017 [96]	ESTD	38/38	Esophagus	39.0/36.0	−	38.0/48.0 (*p* = 0.006)	23/17 (*p* < 0.001)	100/100	0/7.9
Harada et al., 2019 [99]	PCM	46/45	Colorectum	32.5/34.0	−	29.5/41.0 (*p* < 0.001)	20.1/16.3 (*p* < 0.001)	100/100	8.7/8.9
Takezawa et al., 2019 [97]	PCM	280/263	Colorectum	35.3/35.7	−	69.5/78.7	23.5/20.9 (*p* < 0.001)	100/96 (*p* < 0.001)	3.9/4.9
Harada et al., 2018 [103]	PCM	48/48	Stomach	−	34.0/32.5	27.5/41.0 (*p* < 0.001)	22.5/17.3 (*p* < 0.001)	100/100	8.3/6.3
Sakamoto et al., 2017 [104]	PCM	73/53	Colorectum	27/25	−	−	19/14 (*p* = 0.03)	100/92 (*p* = 0.03)	1.4/4.1
Kanamori et al., 2017 [105]	PCM	47/49	Colorectum	26/30	−	77/85	14.3/11.8	100/88 (*p* = 0.015)	10.6/24.5 (*p* = 0.018)

PCM, pocket-creation method; ESTD, endoscopic submucosal tunnel dissection; ESD, endoscopic submucosal dissection.

## Data Availability

Not applicable.
